# Maintaining the balance of TDP-43, mitochondria, and autophagy: a promising therapeutic strategy for neurodegenerative diseases

**DOI:** 10.1186/s40035-020-00219-w

**Published:** 2020-10-30

**Authors:** Chunhui Huang, Sen Yan, Zaijun Zhang

**Affiliations:** 1 Institute of New Drug Research, Guangdong Province Key Laboratory of Pharmacodynamic, Constituents of Traditional Chinese Medicine and New Drug Research, College of Pharmacy, Jinan University, Guangzhou, 510632 China; 2 Ministry of Education CNS Regeneration Collaborative Joint Laboratory, Guangdong-Hongkong-Macau Institute of CNS Regeneration, Jinan University, Guangzhou, 510632 China

**Keywords:** TDP-43, Mitochondria, Autophagy/mitophagy, Neurodegeneration

## Abstract

Mitochondria are the energy center of cell operations and are involved in physiological functions and maintenance of metabolic balance and homeostasis in the body. Alterations of mitochondrial function are associated with a variety of degenerative and acute diseases. As mitochondria age in cells, they gradually become inefficient and potentially toxic. Acute injury can trigger the permeability of mitochondrial membranes, which can lead to apoptosis or necrosis. Transactive response DNA-binding protein 43 kDa (TDP-43) is a protein widely present in cells. It can bind to RNA, regulate a variety of RNA processes, and play a role in the formation of multi-protein/RNA complexes. Thus, the normal physiological functions of TDP-43 are particularly important for cell survival. Normal TDP-43 is located in various subcellular structures including mitochondria, mitochondrial-associated membrane, RNA particles and stress granules to regulate the endoplasmic reticulum–mitochondrial binding, mitochondrial protein translation, and mRNA transport and translation. Importantly, TDP-43 is associated with a variety of neurodegenerative diseases, including amyotrophic lateral sclerosis, frontotemporal dementia and Alzheimer's disease, which are characterized by abnormal phosphorylation, ubiquitination, lysis or nuclear depletion of TDP-43 in neurons and glial cells. Although the pathogenesis of TDP-43 proteinopathy remains unknown, the presence of pathological TDP-43 inside or outside of mitochondria and the functional involvement of TDP-43 in the regulation of mitochondrial morphology, transport, and function suggest that mitochondria are associated with TDP-43-related diseases. Autophagy is a basic physiological process that maintains the homeostasis of cells, including targeted clearance of abnormally aggregated proteins and damaged organelles in the cytoplasm; therefore, it is considered protective against neurodegenerative diseases. However, the combination of abnormal TDP-43 aggregation, mitochondrial dysfunction, and insufficient autophagy can lead to a variety of aging-related pathologies. In this review, we describe the current knowledge on the associations of mitochondria with TDP-43 and the role of autophagy in the clearance of abnormally aggregated TDP-43 and dysfunctional mitochondria. Finally, we discuss a novel approach for neurodegenerative treatment based on the knowledge.

## Background

Neurodegenerative diseases are a group of clinically heterogeneous diseases characterized by the progressive loss or dysfunction of neurons in the central nervous system (CNS) or the peripheral nervous system, including Alzheimer’s disease (AD), Parkinson’s disease (PD), Huntington’s disease (HD), amyotrophic lateral sclerosis (ALS), and frontotemporal dementia (FTD) [1]. Although considerable progress has been made in understanding the mechanisms underlying the transition from pathological changes in brain to neurodegenerative changes, treatments for these diseases have shown limited effectiveness. A common feature of these neurodegenerative diseases is the deposition of abnormally aggregated proteins in extracellular or intracellular inclusions [2, 3].

The discovery of transactive response DNA-binding protein 43 kDa (TDP-43) as the major component of ubiquitin-positive neuronal inclusion bodies is a milestone in understanding the pathogenesis of ALS [4]. In the past decade, TDP-43, encoded by the *TARDBP* gene, was revealed as a key player in the pathogenesis of various neurodegenerative diseases [5, 6]. Since then, rapid progress has been made in understanding the physiological functions of TDP-43 and its role in neurodegeneration [7]. For example, mutations in the TDP-43 gene can cause ALS, and a direct link has been established between TDP-43 and neurodegenerative diseases [8–10]. The neurodegenerative diseases associated with the abnormal aggregation of TDP-43 are collectively referred to as “TDP-43 proteinopathy” [5]. Increasing evidence has suggested that the pathological TDP-43 interferes with multiple pathways including RNA metabolism, protein translation, stress-induced response, autophagy, endocytosis, ubiquitin-proteasome system, and mitochondrial function [11–17]. Although the neurotoxicity of the pathological TDP-43 protein has not been fully elucidated [18–20], the presence of pathologically related TDP-43 inside or outside the mitochondria and the functional participation of TDP-43 in the regulations of mitochondrial morphology, transport and function suggest that mitochondria may be a therapeutic target for TDP-43 proteinopathy [5, 17].

Mitochondria play a vital role in cell bioenergy and apoptosis [21]. Damage to mitochondrial function and cell energy production leads to irreversible cell death. In recent years, the function of mitochondria has become an important topic in research and development of therapeutic drugs for various neurological diseases including ALS, PD, and AD [22]. The structure, function, and localization of mitochondria are closely related to neurodegenerative diseases, and defects in mitochondrial dynamics can contribute to neuronal diseases [23]. Mitochondria have independent genomes and provide oxygen consumption-driven synthesis of ATP (via oxidative phosphorylation, OXPHOS) [24]. However, as a by-product of normal breathing, mitochondria also produce toxic reactive oxygen species (ROS). In addition, the mitochondrial genome accumulates mutations during replication, which ultimately affects the efficiency of OXPHOS. The lack of mitochondria, excess ROS, or both, are likely to be the driving forces of aging, as they reduce the adaptability of cells, cause damage to other organelles, and cause mutations in the nuclear genome [25, 26]. In addition, mutant TDP-43 can damage mitochondrial dynamics, and the overexpression of TDP-43 can cause abnormal aggregation of mitochondria and loss of their normal functions, leading to progressive loss of neurons [27–29]. Moreover, inhibiting the mitochondrial localization of TDP-43 can block the TDP-43-induced mitochondrial dysfunction [17, 30], suggesting that the removal of abnormally aggregated TDP-43 and dysfunctional or damaged mitochondria and restoring the interaction of TDP-43 with mitochondria may be an effective way to treat neurodegenerative diseases.

Autophagy is a basic physiological process that maintains the homeostasis of cells [31] by clearing abnormally aggregated cytosolic proteins and damaged organelles. Autophagy involves a series of sequential events, including double-membrane formation, elongation, vesicle maturation, and ultimately delivery of target substances to lysosomes for degradation and recycling as an energy source [32]. When abnormal or misfolded proteins accumulate in the cytoplasm, nucleus, and extracellular envelopes, they can cause organelle damage and synaptic dysfunction in the nervous system; and autophagy has a regulatory effect on these proteinopathies, including a variety of neurodegenerative diseases [31]. The elimination of TDP-43 by autophagy has received increasing attention [33]. Autophagy can occur as a common phenomenon, such as when cells mobilize energy reserves or lack nutrition, or it can specifically target intracellular components such as abnormally aggregated TDP-43 protein and dysfunctional or damaged mitochondria to maintain cell survival [34, 35]. Thus, research on the neuroprotective effects of autophagy may lead to the development of disease-modifying therapies for neurodegenerative diseases [36, 37].

**Fig. 1:**
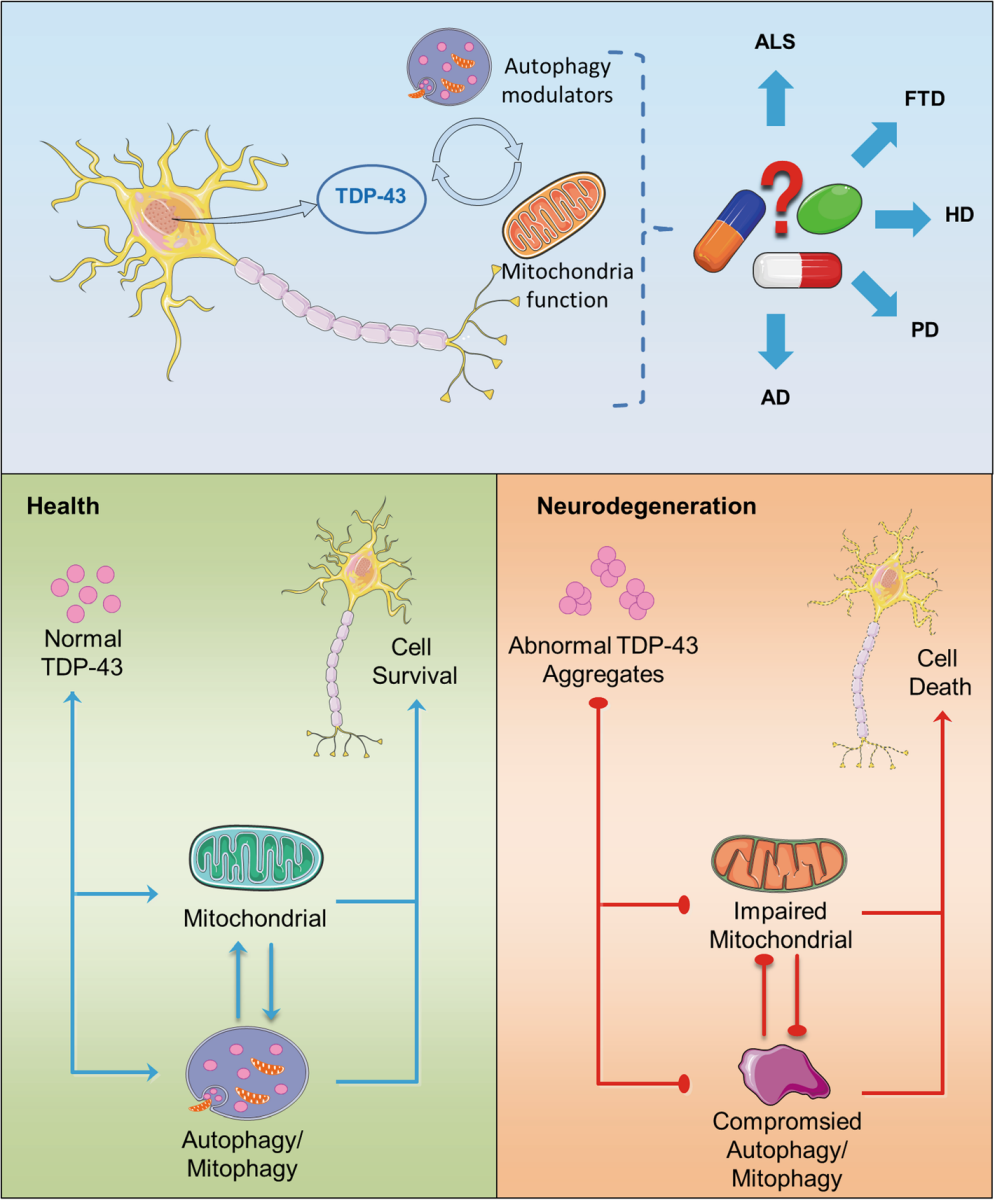
The balance of TDP-43, mitochondria, and autophagy/mitophagy. In the healthy state, TDP-43 promotes mitochondrial biogenesis, mitochondria provide energy for the process of autophagy, and autophagy clears abnormal TDP-43 and damaged mitochondria. In neurodegenerative diseases, TDP-43 aggregates abnormally, impairing mitochondria and autophagy. Due to the autophagy dysfunction, the accumulated TDP-43 aggregates and damaged mitochondria cannot be cleaned up normally, leading to neuronal death.

In summary, the combination of abnormal TDP-43 aggregation, mitochondrial dysfunction, and insufficient autophagy may lead to a variety of aging-related pathologies. In this review, we first summarize current knowledge on the associations between TDP-43 and mitochondria. Then, we describe the role of autophagy in clearing abnormally aggregated TDP-43 and dysfunctional or damaged mitochondria. Finally, we briefly discuss how the studies of TDP-43 proteinopathy, mitochondrial dysfunction, and autophagy may provide a novel approach for treatment of neurodegenerative diseases (Fig. 1).

## Main text

### TDP-43

#### Structure and function of TDP-43

**Fig. 2:**
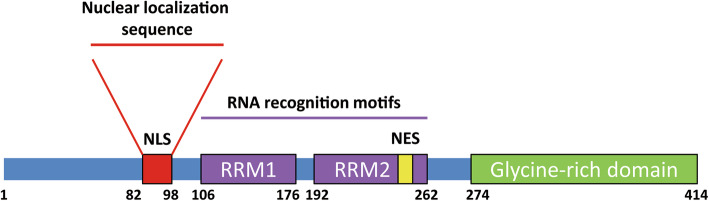
The structure of TDP-43. TDP-43 protein contains a nuclear localization sequence (NLS), two RNA recognition motifs (RRMs), a nuclear export sequence (NES), and a glycine-rich domain.

TDP-43 is a DNA- and RNA-binding protein that belongs to the heterogeneous nuclear ribonucleoprotein family [38]. It consists of 414 amino acids, is encoded by the *TARDBP* gene (chromosome 1p36.2), and has a relative molecular mass of approximately 43000 daltons [39]. TDP-43 contains four functional regions from N- to C-terminus: a nuclear localization sequence (NLS), two RNA recognition motifs (RRMs, RRM1 and RRM2), a nuclear export sequence (NES), and a glycine-rich domain [40] (Fig. 2). Among them, the NLS and NES regulate the shuttle of TDP-43 between nucleus and cytoplasm; RRM1 and RRM2 participate in DNA- or RNA-binding; the glycine-rich domain regulates the splicing activity of TDP-43 and its interactions with other cytokines or organelles, and contains occurrence of most of the disease-related mutations [41, 42].

TDP-43 was originally identified as a transcriptional factor that suppresses the transcription of human immunodeficiency virus 1 [43], and later was proved to be a pre-mRNA splicing regulator [44]. Under normal conditions, TDP-43 is widely present in human and rodent tissues and organs including the heart, lung, liver, spleen, kidney, muscle, and brain [44]. It is mainly located in the nucleus and is capable of nucleus-cytoplasm shuttle [4, 45]. About 30% of TDP-43 protein can be found in the cytoplasm, and its nuclear efflux is regulated by activity and pressure [46, 47]. TDP-43 can bind to RNA and regulate RNA synthesis, splicing, stability and transport, thereby affecting a variety of cellular processes [48]. It also plays a role in the formation of multi-protein/RNA complexes, participates in the regulation of microRNAs and biogenesis, and binds to DNA to inhibit gene transcription [13, 49, 50]. TDP-43 in the cytoplasm can interact with subcellular compartments such as the endoplasmic reticulum (ER) [51, 52], mitochondria [17, 27], mitochondrial-associated membrane (MAM) [53], RNA granules [54], and stress granules [14, 55] to regulate the ER-mitochondrial binding, mitochondrial protein translation, and mRNA transport and translation. Therefore, normal physiological functions of TDP-43 are particularly important for cell survival.

#### Key roles of TDP-43 in neurodegenerative diseases

TDP-43 protein is the main component of tau-negative and ubiquitin-positive inclusion bodies in cortical neurons of frontotemporal lobar degeneration and spinal motor neurons of ALS [4]. TDP-43 aggregation and neuropathology have been observed in a series of unique neurodegenerative diseases, collectively referred to as TDP-43 proteinopathy [18, 56]. TDP-43 proteinopathy occurs through the characteristic histopathological transformation of TDP-43 in the disease [57]. In the pathological state, the TDP-43 proteinopathy occurs through two pathological changes, gain of function and loss of function, which involve phosphorylation, ubiquitination, lysis, reduced solubility, and ectopic cytoplasm expression [57, 58]. The gain of function refers to the cytotoxicity of TDP-43 under abnormal conditions [59]. For example, TDP-43 phosphorylation and ubiquitination are the main pathological changes in patients with TDP-43 proteinopathy, which increase the formation of insoluble inclusions, interfere with the normal function of TDP-43 and lead to a cytotoxic form of TDP-43. In addition, as the C-terminal fragment of TDP-43 has a similar sequence to prion protein, the spread of this toxic peptide in adjacent neurons may also be pathogenic. On the other hand, loss of function refers to the weakening or disappearance of normal functions of TDP-43 after structural change, resulting in abnormal neuronal function including impaired protein degradation, changes in TDP-43-related splicing events, nuclear transport defects, loss of TDP-4 automatic adjustment, and the enhancement of TDP-43 self-interaction [59].

At present, the pathogenesis of TDP-43-associated pathology is still unclear, but its importance in neurodegenerative diseases is self-evident and needs further investigation [6, 60]. Therefore, it is necessary to further study the functions of TDP-43 in order to develop therapeutic strategies that can increase the TDP-43 activity or prevent the TDP-43 aggregation to reduce the potential toxic effects, thereby preventing disease onset or progression.

### Mitochondria and TDP-43

#### Mitochondria and neurodegenerative diseases

Mitochondria have been extensively proven to participate in the basic processes of the nervous system such as energy and intermediate metabolism, calcium homeostasis, and apoptosis [61]. The CNS functions depend largely on effective mitochondrial function because of the high energy demand of brain tissue. It is well known that mitochondria are the powerhouses of cells that produce adenosine triphosphate (ATP), and are the main energy-generating system in most eukaryotic cells [61]. In the cell, energy is produced in the form of ATP, mainly through OXPHOS of mitochondria under aerobic conditions and through anaerobic glycolysis under anaerobic conditions [62]. However, as a by-product of normal breathing, mitochondria also produce ROS that must be detoxified [25]. In addition, the mitochondrial genome accumulates mutations during replication, which ultimately affects the efficiency of OXPHOS [26]. Mutations in mitochondrial DNA (mtDNA), the generation and presence of ROS, and environmental factors may cause energy failure and neurodegenerative diseases including PD, AD, HD and ALS [63].

In addition to the role in chronic aging, the mitochondria also mediate acute cell death. As mitochondria age in the cells, they gradually become inefficient and potentially toxic, and an acute injury can trigger the permeability of mitochondrial membrane, which causes apoptosis or necrosis [24]. Mitochondrial morphological changes manifest as mitochondrial fragmentation and damaged inner membrane structure, which have been increasingly reported as the main early features of major neurodegenerative diseases including ALS and AD [64]. More importantly, the accumulation of misfolded proteins due to gene mutations or abnormal protein homeostasis is a common pathological feature of many neurodegenerative diseases [65]. The binding of misfolded proteins to mitochondria can cause mitochondrial dysfunction, leading to the progressive degeneration of neurons. Previous studies have shown that the mutant TDP-43 damages the mitochondrial dynamics, which causes the abnormal aggregation and a loss of normal function of mitochondria, resulting in a progressive loss of neurons [27–29]. Moreover, recent evidence has shown that inhibiting the mitochondrial localization of TDP-43 can block the toxicity of TDP-43, suggesting that the removal of abnormally aggregated TDP-43 and dysfunctional or damaged mitochondria, and suppressing the TDP-43 interaction with mitochondria may be an effective way to treat neurodegenerative diseases [17, 30]. In the following, we mainly discuss the role of TDP-43 in mitochondrial abnormalities, which are a pathological feature of various neurodegenerative diseases.

#### TDP-43–mitochondrial association

The pathological TDP-43 interferes with multiple mitochondrial pathways, including mitochondrial fission and fusion dynamics, mitochondrial transport, bioenergetics, and mitochondrial quality control, all being critical to the survival of nerve cells [5]. The TDP-43 proteinopathy and mitochondrial abnormalities have gained increasing attention in designing novel treatment approaches for neurodegenerative diseases. Therefore, understanding the internal connection and interaction between TDP-43 and mitochondria is currently a fundamental issue.

The connection between TDP-43 and mitochondria has been proved [5]. It has been shown that TDP-43 exists in the ER [51, 52], mitochondria [17, 27], MAM [53], RNA granules [54], and stress granules [14, 55] to regulate the ER-mitochondria binding, mitochondrial protein translation, and mRNA transport and translation. In yeasts, human wild-type TDP-43 is localized in mitochondria and affects the respiration capacity and function of the electron transport chain [66]. The human wild-type and mutant TDP-43 are also located in the mitochondria of mouse motor neurons, where they activate the mitochondrial phagocytosis and alter the mitochondrial function [67]. Moreover, exogenously expressed wild-type or ALS-related mutant TDP-43 has been detected in the mitochondrion-enriched fraction of NSC-34 motor neuron-like cells, and overexpression of TDP-43 and its C-terminal fragment results in mitochondrial damage [68]. Wang *et al.* [27] have demonstrated that TDP-43 co-localizes with mitochondria in motor neurons, and the ALS-related mutants have enhanced co-localizations. At the same time, co-expression of the mitochondrial fusion protein mitofusin 2 (Mfn2) can eliminate the TDP-43-induced abnormalities of mitochondrial kinetics and mitochondrial dysfunction. These results indicate that the mutant TDP-43 impairs mitochondrial dynamics by enhancing mitochondrial localization, resulting in mitochondrial dysfunction. Furthermore, in HEK293 cells, human and mouse brains, and spinal cord tissues, at least a portion of TDP-43 is located in the mitochondrial intima and contains some putative mitochondrial introduction sequences [17]. The inhibition of TDP-43 mitochondrial localization eliminates the wild-type and mutant TDP-43-induced mitochondrial dysfunction and neuronal loss, and improves the phenotypes of transgenic mice with mutant TDP-43 [17]. In a hemizygous transgenic mouse model expressing the disease-causing human TDP-43 M337V mutant, the cortical neurons are affected by cytoplasmic TDP-43 mislocalization, mitochondrial dysfunction, and neuronal loss [30]. Interestingly, a TDP-43 mitochondrial localization inhibitory peptide eliminates the accumulation of cytoplasmic TDP-43, restores mitochondrial function, prevents neuron loss, and reduces motor coordination and cognitive deficits [30].

However, controversy arose in a subsequent study, which did not found any defects in mitochondrial bioenergy function tested in TDP-43 mutants or a correlation between TDP-43 and respiratory chain dysfunction [69]. In contrast, recent studies have reported that the 35 kDa truncated form of TDP-43 is limited to the mitochondrial inner membrane space, while the full-length form is also located in the mitochondrial matrix of NSC-34 cells [70]. Interestingly, the full-length form may significantly affect mitochondrial metabolism and morphology by inhibiting the expression of Complex I subunits encoded by mitochondrial transcribed mRNA [70]. Additional evidence has shown that in the mouse model of motor neuron disease, full-length TDP-43 has increased associations with mitochondria, while blocking the TDP-43/mitochondrial interaction improves motor dysfunction [71]. Moreover, recent studies have reported that cytoplasmic mislocalization and mitochondrial localization of TDP-43 are common features in normal elderly mice [72].

The co-localization of TDP-43 and mitochondria has been demonstrated using different approaches, and suggests that TDP-43 plays a vital role in the damage of the structure and function of mitochondria. Accordingly, understanding how TDP-43 co-localizes with mitochondria and results in abnormal mitochondrial structure and function has become a particularly critical issue.

#### TDP-43 mitochondrial localization pathway

TDP-43 has various isoforms generated by caspases, which can recognize the endogenous cleavage site in the protein and generate two C-terminal fragments, namely, 25-kDa and 35-kDa fragments [4, 73]. Unlike the 25-kDa fragment, the 35-kDa TDP-43 retains the RRM1 and RRM2 sequences responsible for interacting with RNA, thereby maintaining the ability to regulate RNA maturation [74]. However, this truncated form has a defective nuclear localization signal and accumulates in the cytoplasm, easily forming aggregates [75]. According to the study by Wang et al. [17], the entry of TDP-43 into mitochondria is driven by three internal protein motifs (M1, M3, and M5) that are rich in hydrophobic amino acids, while deletion of these motifs suppresses the mitochondrial import of exogenously expressed TDP-43. The 35-kDa fragment also lacks the M1 sequence that was reported to drive TDP-43 mitochondrial localization, while retaining the M3 and M5 putative signals. Moreover, mitochondrial chaperones that interact with TDP-43, including voltage-gated anion channel 1 and prohibitin 2, a key mitochondrial receptor, are another mechanism underlying the mitochondrial localization of TDP-43 [71]. To date, although there are few data on how TDP-43 accumulates in the mitochondria, it is an indisputable fact that they have a close relationship. Therefore, a novel therapeutic strategy may be proposed to prevent TDP-43 entry or attachment to mitochondria.

#### TDP-43 causes damage to mitochondria

Although the pathological mechanism of TDP-43 proteinopathy is unclear, pathologically related TDP-43 has been shown to exist inside and outside the mitochondria, and participate in the regulation of mitochondrial morphology, transport, and function, suggesting that mitochondria may be a target of TDP-43 proteinopathy. Numerous studies have demonstrated that pathological TDP-43 interferes with multiple mitochondrial pathways including mitochondrial fission and fusion kinetics, mitochondrial transport, bioenergetics, and mitochondrial quality control [5].

### TDP-43 and mitochondrial fission and fusion dynamics

Studies have shown that the mitochondrial fission and fusion dynamics are essential for almost all aspects of mitochondrial function including respiratory complex assembly [76], ATP production [77], Ca^2+^ homeostasis [78], and ROS production [76]. Mitochondrial fission and fusion are strictly controlled by several key regulatory factors, including dynamin-related protein 1 (Drp1) and its recruitment factors for mitochondria such as Mff and Fis1 [79], Mfn1, Mfn2, and optic atrophy protein 1 (OPA1) [23]. It is worth noting that the morphological changes seen in the TDP-43 experimental model are consistent with the reported changes in the expression of mitochondrial fission and fusion regulators such as Drp1, Fis1, Mfn1 and OPA1 [71, 80]. Although the mechanism by which TDP-43 regulates mitochondrial dynamics is still elusive, previous studies have reported that the mutant TDP-43-induced mitochondrial fragmentation can be alleviated by overexpression of Mfn2, suggesting that the Mfn2-dependent fusion may be involved [71]. Consistently, a recent study showed that there is a physical interaction between TDP-43 and Mfn2. However, the overexpression of wild-type TDP-43 in the brain increased the expression of Mfn2, rather than reducing it, which indicates that the wild-type and mutant TDP-43 may interfere with mitochondrial dynamics through different mechanisms.

In addition to the neurofibrillary tangles and senile plaques, cytoplasmic TDP-43 inclusions are also considered to be a possible proteinopathy in AD patients [81]. Although there are limited studies on TDP-43 and mitochondrial dynamics in AD-related experimental models, recent studies have reported that TDP-43 increases the expression of Mfn2, and overexpression of wild-type TDP-43 causes mitochondrial enlargement and swollening in the hippocampal neurons of APP/Parkin–presenilin1 (PS1) transgenic mice [71]. However, it is unclear how these findings are related to previously reported mitochondrial embrittlement and reduced Mfn2 expression in AD patients and experimental models, but the co-presence of the two is a pathological feature of AD and many other neurodegenerative diseases [82, 83]. The synergistic effect of TDP-43 and other protein diseases on mitochondrial dynamics warrants detailed studies in the future.

### TDP-43 and mitochondrial trafficking

Synaptic loss is an important pathological feature preceding neurodegeneration. The failure of correct positioning of mitochondria at the end of dendrites or axons has long been considered to be associated with neurodegenerative diseases and to be a potential cause of synaptic loss [22]. In addition to the altered mitochondrial morphology, impaired mitochondrial transport also occurs in cell and animal models with TDP-43 aberration [5]. In primary motor neurons, overexpression of wild-type TDP-43 leads to impaired anterograde and retrograde transport of mitochondria in axons and dendrites, which is further exacerbated in the context of ALS-related mutations [27]. Unexpectedly, similar to TDP-43 overexpression, the loss of TDP-43 also reduces mitochondrial transport in axons and dendrites, suggesting that TDP-43-mediated mitochondrial transport may involve different pathways [27]. Importantly, the defects in mitochondrial transport seem to be an early pathological feature of TDP-43 transgenic mice, which precedes the onset of symptoms and even morphological abnormalities [84]. Interestingly, motor neurons with TDP-43 mutations in human-induced pluripotent stem cells show an age-dependent dramatic decrease in the speed of mitochondrial movement at the proximal and distal axons [85]. Conversely, no accumulation of inclusions or phosphorylated TDP-43 has been detected in the cytoplasm [85], which further indicates that mutant TDP-43 may cause mitochondrial toxicity regardless of the proteinopathy.

### TDP-43 and mitochondrial function

TDP-43 has unpredictable effects on mitochondrial function. In experimental models related to TDP-43, mitochondrial OXPHOS defects have been widely reported. In NSC-34 cells overexpressing wild-type or mutant TDP-43, decreases in mitochondrial complex I activity and mitochondrial transmembrane potential have been observed, accompanied by the increased expression of mitochondrial uncoupling protein 2, followed by a decrease in ATP synthesis [67, 86]. Furthermore, full-length mitochondrial internal TDP-43 can be combined with mitochondrial transcribed mRNA encoding the OXPHOS complex I subunit (ND3/6) to specifically impair its assembly and function [70], while truncated TDP-43 without the M1 mitochondrial location sequence has no effect on ND3/6 expression or mitochondrial function [70]. In addition, TDP-43-mutant ALS-derived lymphoblast cell lines exhibit perturbed mitochondrial function, including increased basal oxygen consumption rate and decreased spare respiratory capacity, suggesting impaired energy production capacity of mitochondria [87]. Interestingly, the cytotoxicity of TDP-43 in yeasts can be changed by manipulating mitochondrial function. Specifically, the respiration-related ROS can enhance the toxicity of TDP-43, so activating TDP-43 through respiration makes it more toxic or makes TDP-43 targets more vulnerable [88]. In addition, TDP-43 has been reported to interfere with the ER-mitochondrial association [53, 89], which is important for Ca^2+^ homeostasis, lipid metabolism, autophagy, and even protein transport. This evidence shows that there is an inextricable relationship between TDP-43 and mitochondria, which provides necessary information for treating TDP-related neurodegenerative diseases. Considering the ability of TDP-43 to regulate mitochondrial function, clearing abnormal TDP-43 or blocking the interaction between TDP-43 and mitochondria may lead to unpredictable benefits to the disease. Simultaneously, mitochondria have a regulatory function on TDP-43 toxicity, which suggests that dealing with mitochondrial dysfunction or abnormal mitochondria is one of the strategies for treating TDP-43-related diseases.

### Autophagy clearance of misfolded TDP-43 and abnormal mitochondria

#### Autophagy and neurodegenerative diseases

Autophagy is a catabolic process that acts on all cells of the body and removes toxic and damaged substances through the degradation process. The main regulatory event in the process of autophagy induction is the triggering of the interaction of the complex with nutrient-sensitive mTOR kinase and energy-sensitive AMP-activated protein kinase (AMPK) and involves more than 35 autophagy-related genes (*atg*) [90, 91]. Specifically, a decrease in cellular energy activates AMPK, phosphorylating Unc-51-like autophagy activating kinase 1 (ULK1) at serine 317 and serine 777 [90]. These phosphorylation events in turn activate ULK1, which initiates autophagy [92]. Conversely, the presence of nutrients activates mTORC1 (through amino acid binding), thereby phosphorylating ULK1 on serine 757, leading to the inhibition of autophagy [90]. Therefore, both nutrition- and energy-sensing mechanisms can prevent the occurrence of autophagy, a process in which cells degrade and restore cellular components through lysosomes to balance energy sources and structural units, thereby maintaining cell homeostasis and function [93]. Therefore, the reduction of autophagy promotes the accumulation of substances that otherwise are normally removed from cells, and adversely affects cell survival.

Autophagy is necessary to maintain the normal function of the CNS to avoid accumulation of misfolded and aggregated proteins [33]. Extracellular or intracellular inclusions contain abnormally aggregated proteins that are easily aggregated and are a common feature of many neurodegenerative diseases [2, 3]. Consistently, impaired autophagy is associated with the pathogenesis of various neurodegenerative diseases [31]. Autophagy and proteasome are considered to be the main ways to promote protein degradation. Once the autophagy function is impaired, it will damage the homeostasis and physiological functions of cells [94]. For example, the elimination of TDP-43 by autophagy can protect against a variety of neurodegenerative diseases [33]. Furthermore, the neuron-specific deletion of essential autophagy genes (*Atg5* and *Atg7*) inhibits autophagy and promotes the neurodegenerative phenotype, which is characterized by axonal degeneration and accumulation of aggregation-prone proteins in the neuronal cytoplasm [95, 96].

In addition to the removal of abnormally aggregated proteins, the most relevant function of autophagy is to clean up damaged organelles, such as dysfunctional mitochondria or damaged mitochondria [34, 35, 97]. In particular, mitophagy, which is the targeted phagocytosis and destruction of mitochondria by autophagy devices, is generally considered to be the mechanism primarily responsible for mitochondrial quality control [98]. Interestingly, oxidative stress is an effective regulator of autophagy, suggesting that functional interactions may occur between the lysosome and mitochondrial pathways [99–101]. Moreover, recent observations indicate that TDP-43 is not just a passive substrate for autophagy; instead, it seems to be actively involved in autophagy activation [102]. Inadvertently, it has been observed that autophagy not only clears TDP-43 and damaged or dysfunctional mitochondria but can also be reversely regulated by them. Therefore, it can be speculated that there is a balance among mitochondria, TDP-43, and autophagy, which is largely an entry point for the treatment of neurodegenerative diseases.

#### Autophagy and TDP-43

##### Regulation of TDP-43 by autophagy

Numerous evidence has indicated that autophagy has a clearing effect on TDP-43. Studies have shown that the autophagy-related proteins such as LC3 and p62/SQSTM1 co-localize with TPD-43 aggregates, proving that autophagy is necessary for preventing accumulation of TDP-43 aggregates [103, 104]. In addition to the autophagy-related proteins p62/SQSTM1 and LC3, the autophagy regulators vasolin-containing protein and OPTN are co-localized with TDP-43 inclusions in the spinal motor neurons of patients with sporadic ALS [105, 106], which further validates the effect of autophagy on TDP-43. Therefore, autophagy clearance is directly related to the pathological accumulation of TDP-43 aggregates.

TDP-43 loss-of-function increases the activity of transcription factor EB (TFEB), a major regulator of lysosomal biogenesis and autophagy, and prevents the autophagosome-lysosomal fusion, while inhibition of mTORC1 signaling by rapamycin exacerbates the neurodegenerative phenotype in the *Drosophila* model of TDP-43 deficiency [15]. Furthermore, inhibition of autophagy with 3-methyladenine can promote the accumulation of full-length TDP-43 and its degradation products of 35 kDa and 25 kDa in N2a and SH-SY5Y cells [107]. Similar results were observed in HEK-293 cells overexpressing GFP-TDP-43 WT or GFP-TDP-25 kDa fragments. On the other hand, when autophagy is induced in N2a and SH-SY5Y cells by rapamycin treatment, the degradation of different forms of TDP-43 is enhanced. These findings suggest that autophagy induction may be an effective therapeutic target for TDP-43 proteinopathy.

The level of TDP-43 protein controlled by autophagy can be jointly regulated by heat shock protein (HSP). For instance, induction of autophagy by downregulating HSP-90 or cell division cycle 37 (CDC37) promotes the degradation of HSP-90, CDC37 and TDP-43 protein complexes [108]. Same results have been observed in the motor neuron-like cell lines NSC-43 and SH-SY5Y [109–111]. Specifically, overexpression of small HspB8 induces the degradation of TDP and its truncated form by increasing autophagy [110]. Subsequently, it was found that the upregulation of HspB8 induced by colchicine and doxorubicin enhances the expression of *tfeb*, *p62*/*sqstm1* and *lc3*, indicating that HspB8 may activate autophagy [111]. Previous studies have shown that the C terminus of Hsp70-interacting protein (CHIP) is a key regulator of UPS and ALP [112]. The differential expression of HspBP1 plays an important role in the elimination of misfolded proteins in neurons and astrocytes. Overexpression of HspBP1 inhibits the activity of CHIP and causes abnormal aggregation of misfolded proteins, while the highly active CHIP contributes to the elimination of mutant TDP-43 [113].

Together, these studies show that autophagy affects the aggregation of TDP-43 *in vitro* and *in vivo*, and the control of TDP-43 protein level is critically essential. Autophagy is crucial for the occurrence and development of TDP-43-related neurodegenerative diseases. Regulation of autophagy toward the degeneration of TDP-43 protein will become a potential strategy for the treatment of these diseases.

##### Regulation of autophagy by TDP-43

Autophagy regulates the accumulation of TDP-43, and TDP-43 is involved in the regulation of autophagy. In the mRNA hybridization experiment, researchers found that TDP-43 bond to atg7 mRNA through its RRM1 domain [102]. The downregulation of TDP-43 reduced the level of atg7 mRNA, accompanied by decreased levels of the ATG7 protein, LC3-II, p62/SQSTM1, autophagosomes, and ubiquitinated inclusions, all of which are indicators of impaired autophagy [102].

In addition, TDP-43 is required to maintain the mTOR activity by stabilizing the mRNA level of RAPTOR, and mTOR further maintains the cytoplasmic position of TFEB by phosphorylating TFEB [15]. Therefore, the downregulation of TDP-43 reduces the mTOR-dependent phosphorylation of TFEB and induces TFEB translocation to the nucleus, thereby increasing the expression of *lamp1*, *lamp2*, *atg5*, *beclin-1*, *cathepsinL* and other autophagy-related genes [15]. Furthermore, the accumulation of LC3-II, lysosomes and autophagosomes, and the downregulation of TDP-43 induce the accumulation of p62/SQSTM1, indicating that the TDP-43 is required for autophagosome-lysosomal fusion because TDP-43 contributes to the stabilization of dynactin 1 mRNA and dynactin 1 is involved in the autophagic body-lysosomal fusion. Therefore, downregulation of TDP-43 reduces the level of dynactin 1 protein, thus impairing the autophagosome-lysosome fusion [88]. In short, the lack of TDP-43 eventually damages the process of autophagy flux.

Latest research shows that the chaperone-mediated autophagy (CMA) degrades TDP-43 protein and can be affected by TDP-43 aggregation [114]. The aggregated form of TDP-43 can interact with Hsc70, co-localize with Lamp2A, and upregulate the levels of these molecules to enhance CMA, a lysosomal degradation pathway [114]. It has been speculated that TDP-43 is not only a CMA substrate, but the conversion of its physiological and pathological forms is controlled by CMA, and TDP-43 polymerization affects the performance of CMA.

In summary, the binding of TDP-43 aggregates results in the loss of TDP-43 function [115, 116], and the lack of TDP-43 activity may increase cell stress by inhibiting autophagy. Considering this scenario, the lack of autophagy will further enhance the accumulation of TDP-43 aggregates, resulting in increased cell stress, cell death, and neurodegeneration. These findings have been confirmed in a transgenic mouse model overexpressing the 25-kDa TDP-43 fragment in the brain and spinal cord, which showed a reduction in both autophagy and cognitive changes, accompanied by a decline in autophagy function with decreased autophagy markers such as atg3, Atg7, LC3-II, p62/sqstm1 and Beclin 1 [117]. This provides a new insight into the maintenance of the TDP-43–autophagy balance to fight neurodegenerative diseases.

#### Autophagy and mitochondria

##### Mitophagy

Mitochondria are not only energy generators necessary for tissue homeostasis, but also channels for programmed apoptosis and necrotic cell death. Strict control of the quality and quantity of mitochondria is essential for normal functions of these organelles [98]. In neurons, mitophagy effectively removes damaged mitochondria, playing a fundamental role in mitochondrial and metabolic homeostasis, energy supply, neuron survival, and health [118]. Abnormal mitochondrial accumulation in AD is thought to be associated with mitophagy deficits. Research in the past few decades has shown that these neurodegenerative diseases are related to mitochondrial dysfunction and impaired mitochondrial phagocytosis, which lead to the accumulation of protein aggregates and ultimately to neurodegeneration [119].

The most studied mitophagy pathway is mediated by the PTEN-induced putative protein kinase 1 (PINK1) and Parkin. Mutations in *PINK1* and *PARK2* contribute to obvious mitochondrial dysfunctions, leading to degeneration of muscles and neurons [120]. Abnormal mitochondrial accumulation in AD is thought to be caused by multiple mechanisms of mitophagy defects, such as the impaired PS1/γ-secretase–amyloid precursor protein intracellular domain–PINK1 transcription axis [121]. In PD, *PARK6* (coding PINK1) and *PARK2* (coding Parkin) gene mutations will cause 5% of familial PD [122]. In addition, many genes related to ALS and FTD encode proteins involved in mitophagy/selective autophagy, including OPTN, TBK1, p62, and receptor interacting protein kinase 1, although their pathological contributions remain to be clarified [123–126]. Moreover, the GAPDH-mediated mitophagy damage caused by mutant Htt is related to the pathogenesis of HD [127]. In summary, mitochondrial damage is likely a common phenomenon of neurodegenerative diseases, and dysregulation of mitochondrial clearance may trigger various forms of neurodegeneration.

##### Mitochondria regulate autophagy

Damaged mitochondria are cleared by autophagy. In contrast, normal mitochondria have an extremely important regulatory effect on the induction of autophagy. Mitochondrial energy deprivation is the hub of autophagy induction [128]. In starved cells, the outer membrane of mitochondria participates in autophagosome biogenesis [35]. Specifically, the autophagy markers ATG5 and LC3 transiently localize to mitochondria during autophagy, suggesting that the mitochondria contribute membrane to autophagosomes [129]. Moreover, the autophagy markers ATG14 and ATG5 are localized at the ER-mitochondrial contact site during starvation, and disruption of the mitochondrial/ER connection greatly attenuates starvation-induced autophagy [130]. It is worth noting that the reduction of the ATP:AMP ratio in cells may act as a negative regulator of mTOR through AMPK, or directly through the phosphorylation of ULK1 to activate autophagy [92].

ROS produced in mitochondria were initially considered to be harmful byproducts of oxidative metabolism [99, 131], but recent research has suggested that ROS may participate in the regulation of autophagy pathway [100, 132]. Nutritional starvation leads to the accumulation of H_2_O_2_ in mitochondria through mitochondrial PI3K, which is essential for the induction of autophagy [99]. Exogenous H_2_O_2_ treatment in malignant glioma cells can activate autophagy, and these cells exhibit reduced BCL2 expression and increased BAX levels, leading to the loss of mitochondrial membrane potential and the release of cytochrome c [133]. Concomitantly, the H_2_O_2_ treatment results in increased levels of mammalian Atg6 homologue beclin-1 and decreased mTOR activity, which contribute to the induction of autophagy [134]. The role of O_2_^-^ produced in mitochondria in the regulation of autophagy cannot be ignored [100]. For example, the anticancer agent sodium selenite induces mitochondrial damage and activation of selective autophagy, during which the O_2_^-^ participates in the signal transmission for autophagy activation [135]. Taken together, mitochondria can be regarded as both “victims” of autophagy and regulators of the signaling pathways that ultimately lead to autophagy. However, in TDP-43-related neurodegenerative diseases, research on mitochondrial regulation of autophagy is insufficient, so more studies are needed. In other words, how to maintain the balance between autophagy and mitochondrial regulation has become a new topic for neurodegenerative disease research.

### Therapeutic strategies targeting TDP-43, mitochondria, and autophagy

It is clear that abnormally aggregated TDP-43, damaged mitochondria, and impaired autophagy play important roles in neurodegenerative diseases. Abnormal accumulation of TDP-43 can damage mitochondria and autophagy. Furthermore, regulation of the autophagy pathway by mitochondria is so important that the dysfunction of mitochondria may block normal autophagy. Similarly, damaged mitochondria can interfere with normal TDP-43, while dysfunctional autophagy cannot clean up the accumulated TDP-43 aggregates and the damaged mitochondria. Therefore, it has been widely recognized that targeting TDP-43 [136, 137], mitochondria [138, 139], and autophagy [36, 37] to protect neurons may be a therapeutic strategy. Therefore, maintaining the TDP-43-mitochondria-autophagy balance is a promising way to treat neurodegenerative diseases.

Several small-molecule drugs have been reported to target TDP-43, mitochondria, and autophagy (Table 1), adding to the promising future of treatment of neurodegenerative diseases. Their specific roles have been described in other reviews and will not be described here [138, 140, 141].

**Table 1:** Small molecule compounds targeting TDP-43, mitochondria, and autophagy

Target	Drug	Mechanism	Reference
TDP-43	Mitoxantrone	Reduces the recruitment of TDP-43 from SGs and prevents the formation of TDP-43 aggregates	[142]
	Trimethylamine N-oxide (TMAO)	Enhances TDP-43 LLPS but prevents protein fibrillation in vitro	[143]
	rTRD01	Prevents RNA or DNA from binding to TDP-43 to reduce neuronal toxicity	[144]
	PF 670462	Inhibits casein kinases 1 to reduce TDP-43 phosphorylation and aggregation	[145, 146]
	D4476		
	Olomoucine	Inhibits cyclin-dependent kinase 2 (CDK2) to reduce TDP-43 accumulation in SGs	[147, 148]
	SB 415286	Inhibits glycogen synthase kinase 3β (GSK3β) to reduce TDP-43 accumulation in SGs	
	IGS2.7	Inhibits CK1 to reduce TDP-43 phosphorylation and restores nuclear protein localization to restore TDP-43 homeostasis	[149]
	IGS3.27		
Mitochondria	MitoQ	Mitochondria-targeting antioxidant	[150]
	AICAR	Activates AMPK, which then acts on PGC-1α	[151]
	Nicotinamide mononucleotide (NMN)	Increases NAD^+^ pools and activates mitochondrial unfolded protein response	[152–154]
	Resveratrol	Activates SIRT1, which then acts on PGC-1α	[155, 156]
	Rimonabant	Activates eNOS; increases mitochondrial mtDNA and mRNA	[157]
	Fibrates	Activates AMPK and PGC-1α	[158]
	Recombinant TFAM	Increases respiration and mitochondrial biogenesis	[159, 160]
Autophagy/ Mitophagy	Melatonin	Counteracts oxidative stress, and prevents collapse of mitochondrial membrane potential; accumulates within mitochondria to prevent cardiolipin peroxidation in order to maintain cardiolipin interaction with autophagosomes via LC3II	[161–163]
	Urolithin A	Induces expression of mitophagy proteins, including full-length PINK1, Parkin, OPTN; p-ULK1, LC3B-II, Beclin1, Bcl2L13, AMBRA1, and FUNDC1 in SH-SY5Y cells; induces expression of full-length PINK1 in brain tissues of mice	[164, 165]
	Actinonin	Induces expression of mitophagy proteins, including full-length PINK1, Parkin, OPTN; p-ULK1, LC3B-II, Beclin1, Bcl2L13, AMBRA1, and FUNDC1 in SH-SY5Y cells; induces expression of full-length PINK1 in brain tissues of mice	[164, 166]
	Rapamycin	Induces macro-autophagy by direct binding and inhibition of mTOR; stimulates AMPK; extends lifespan in mice in an ULK1-dependent manner	[167–169]
	Metformin	Induces autophagy/mitophagy via SIRT1, IGF-1, and mTORC1, or via Parkin-mediated mitophagy	[167, 170–172]
	Spermidine	Induces mitophagy through multiple pathways, involving the ATM-PINK1-Parkin pathway, the Nrf2-SKN-1 pathway, and through activation of AMPK and inhibition of mTOR; inhibits EP300; induces BNIP3, CTSL, and ATGs	[173–175]
	Torin1	Induces autophagy by inhibiting the kinase domains of two TORC complexes	[176, 177]
	Lithium	Removes altered mitochondria and protein aggregates	[178]

In addition to a single target, there are drugs that act on multiple targets at the same time, such as melatonin, which can resist oxidative stress and prevent the breakdown of mitochondrial membrane potential [161], and can also promote the basic level of autophagy, thereby maintaining the steady state and survival of neurons [162]. As another example, nicotinamide mononucleotide can activate mitochondrial unfolded protein response through NAD^+^ replenishment [152], accompanied by enhanced autophagy by sirtuin-dependent deacetylation of Atg5, Atg7, and Atg8 [179]. In both *in vitro* and *in vivo* models, lithium prevents most of the pathological changes of ALS through mechanisms such as mitochondrial protection, autophagy induction, mitochondrial autophagy, and mitochondrial biogenesis, all being evidence for targeting both mitochondria and autophagy [178, 180–182].

## Discussion

In this paper, we review crucial roles of TDP-43, mitochondria, and autophagy in neurodegenerative diseases. TDP-43 plays an important role in various DNA/RNA processes. Unfortunately, the accumulation of abnormal TDP-43 can cause severe damage to the mitochondrial morphology, structure and function, as well as autophagy, leading to a variety of neurodegenerative diseases [7]. Subsequently, mitochondrial damage caused by TDP-43 further exacerbates the autophagy disorder. In turn, the autophagy disorder causes a failure to clear the accumulated TDP-43 and abnormal mitochondria, resulting in accumulation of TDP-43 and damaged mitochondria in cells and thus cell death, eventually leading to neurodegenerative diseases.

These correlations have been confirmed in various studies from multiple angles. The most direct evidence is that the pathologically relevant TDP-43 has been repeatedly proven to exist inside or outside the mitochondria, and is functionally involved in the regulation of mitochondrial morphology, transport, and function [5]. In addition, mutant TDP-43 impairs the mitochondrial dynamics, and the overexpression of TDP-43 will cause abnormal aggregation and a loss of function of mitochondria, resulting in the progressive loss of neurons [27–29]. It is worth mentioning that inhibiting the mitochondrial localization of TDP-43 can block its toxicity [17, 30], suggesting that the removal of abnormally aggregated TDP-43 and dysfunctional or damaged mitochondria, and blocking the interaction between TDP-43 and mitochondria may be an effective way to treat neurodegenerative diseases. However, discrepancies exist across studies. For example, TDP-43 is only present in the membrane of HEK293 or HeLa cells associated with mitochondria and in the mouse brain [69]. In contrast, recent studies using NSC-34 cells have reported that the full-length and truncated form of TDP-43 have different presence, with the truncated form restricted to the intermembrane space of mitochondria while the full-length form also localizing in the mitochondrial matrix [70]. Studies using mouse cortex and hippocampal tissues have shown that there is truncated TDP-43, but no full-length form, in the mitochondria [71]. Thus, TDP-43 may exist differently in different cells. In addition, the post-translational modifications may play a crucial role in the accumulation of mitochondrial TDP-43 in disease. Therefore, the development of alternative or novel methods to determine the mitochondrial sublocalization of different forms of TDP-43 warrants further investigation. Furthermore, in patients with TDP-43-related neurodegenerative diseases, it is unclear how TDP-43 binds to mitochondria. Although there is still controversy, all previously published studies unanimously support the direct binding of TDP-43 to mitochondria.

Interestingly, autophagy/mitophagy clears abnormally aggregated proteins and impaired organs, including TDP-43 and abnormal mitochondria [33–35], which suggests that regulating autophagy/mitophagy is an extremely advantageous therapeutic approach. However, the accumulated TDP-43 and damaged mitochondria have an irreversible and disastrous effect on autophagy/mitophagy function [89, 114, 128]. That is, if any part of the balance of TDP-43, mitochondria, and autophagy/mitophagy is affected, a vicious circle will occur. Therefore, a strategy that improves any link or even multiple links may have beneficial effects against the disease.

At present, the TDP-43-targetting drugs mainly work by regulating TDP-43 levels and modifying TDP-43 status (e.g. phosphorylation) to reduce the accumulation of abnormal TDP-43 aggregates [149]. Five broad treatment strategies have been proposed to directly or indirectly affect mitochondria in mitochondrial diseases: repairing or preventing damage to organelles; inducing mitochondrial biogenesis; enhancing organelle quality control by stimulating the degradation of damaged mitochondria or organelle components; manipulating mitochondrial function to induce cell death; and changing mitochondrial signaling pathways or metabolic processes [138]. In addition, there are many potential therapeutic strategies using gene therapy to correct for defective genes or for ectopic expression of mtDNA designed to degrade mutants or alter metabolic proteins [183, 184]. In addition, the autophagy function can be induced to maintain the steady state of proteins and organelles to exert neuroprotection [36].

Although accumulating evidence has shown the protection of regulation of TDP-43 or the mitochondrial or autophagy process against neurodegenerative diseases, it remains to be verified whether multi-step treatment is superior to the single-target treatment. In addition, the mechanism underlying the balance among TDP-43, mitochondria and autophagy remains to be studied. On the one hand, we can clarify the internal connections between TDP-43, mitochondria, and autophagy/mitophagy by establishing more advantageous models such as pig and non-human primate models of neurodegeneration [185, 186]. On the other hand, exploring and discovering new drugs targeting TDP-43, mitochondria and autophagy, either alone or in combination, is a key step that must be taken. Inevitably, it is more helpful to explore the effects of existing drugs that can separately regulate TDP-43, mitochondria, and autophagy in TDP-43-related neurodegenerative diseases on the other two targets.

## Conclusions

In conclusion, it is evident that TDP-43 protein pathology, mitochondrial disorders, and impaired autophagy are common prominent pathological features of major neurodegenerative diseases including ALS, FTD, and AD. Inhibition of mitochondrial localization of TDP-43 is sufficient to alleviate mitochondrial dynamic abnormalities, neuronal loss, and behavioral defects in transgenic mice with different mutant forms of TDP-43. Due to the close relationship between mitochondrial and autophagy functions and TDP-43, and their contributions to the progression of neurodegenerative diseases, we believe that maintaining the balance among TDP-43, mitochondria, and autophagy is a promising strategy for the treatment of neurodegenerative diseases.

## Data Availability

Not applicable.

## Abbreviations

atg: Autophagy-related genes
AD: Alzheimer’s disease
ALS: Amyotrophic lateral sclerosis
AMPK: AMP-activated protein kinase
ATP: Adenosine triphosphate
CDC37: Cell division cycle 37
CMA: Chaperone-mediated autophagy
CNS: Central nervous system
Drp1: Dynamin-related protein 1
ER: Endoplasmic reticulum
Fis1: Fission protein 1
FTD: Frontotemporal dementia
HD: Huntington’s disease
HSP: Heat shock protein
HspB8: Heat shock protein B8
mtDNA: mitochondrial DNA
MAM: Mitochondrial-associated membrane
Mff: Mitochondrial fission factor
Mfn1: Mitofusin 1
Mfn2: Mitofusin 2
NES: Nuclear export sequence
NFT: Neurofibrillary tangle
NLS: Nuclear localization sequence
OPA1: Optic atrophy protein 1
OXPHOS: Oxidative phosphorylation
PD: Parkinson’s disease
ROS: Reactive oxygen species
RRM: RNA recognition motifs
TDP-43: Transactive response (TAR) DNA binding protein 43 kDa
